# Relationship between maternal social support and undernutrition among children under 5 years in Siem Reap Province, Cambodia: the chain mediating roles of household food insecurity and maternal depression

**DOI:** 10.1186/s12889-025-25913-0

**Published:** 2025-12-11

**Authors:** Mengleang Rek, Nabila Ibn Ziyat, Rui Chen, Xixing Xu, Tenbite Daniel Mekonnen, Filimon Asseged Belay, Mohamad Ramadhani Babuhija, Pheng Ngov, Shuai Wang, Fanlei Kong

**Affiliations:** 1 Department of Social Medicine and Health Management, School of Public Health, Cheeloo College of Medicine, Shandong University, Jinan, 250012 China; 2 NHC Key Lab of Health Economics and Policy Research (Shandong University), Jinan, 250012 China; 3 Department of Orthopaedics, The Second Affiliated Hospital of Chongqing Medical University, Chongqing, 401336 China; 4 Department of Pediatric Surgery, Shandong Provincial Hospital Affiliated to Shandong First Medical University, Jinan, 250012 China

**Keywords:** Undernutrition, Stunting, Wasting, Maternal social support, Household food insecurity, Maternal depression

## Abstract

**Background:**

Undernutrition among children under five years (CUFY) is a leading global public health issue worldwide, including in Cambodia. This study aimed to determine the prevalence of undernutrition and the mediating effect of maternal depression and household food insecurity on the association between maternal social support and undernutrition among CUFY in Siem Reap Province, Cambodia.

**Method:**

A semi-structured questionnaire was utilized to survey 450 mothers in Siem Reap Province, Cambodia. Maternal social support, maternal depression, and household food insecurity were assessed separately using the Maternity Social Support Scale (MSSS), Patient Health Questionnaire-2 (PHQ-2), and Household Food Insecurity Access Scale (HFIAS). Undernutrition indicators were evaluated using height-for-age z-score (HAZ) and weight-for-height z-score (WHZ), calculated according to WHO Anthro standards. A chi-square test was employed to illustrate the distribution of the variables. Structural equation modeling was used to analyze the chain-mediating effect of maternal depression and household food insecurity on the relationship between maternal social support and undernutrition among CUFY.

**Result:**

The prevalence of stunting and wasting among CUFY was 34.9% and 15.6%, respectively. Structural equation modeling (SEM) results showed that maternal social support had a negative effect on children’s undernutrition (β = -0.32), indicating that social support is negatively correlated with child undernutrition. Women with higher levels of social support are less likely to have a child suffering from undernutrition compared to women with lower levels of social support. Household food insecurity and maternal depression mediated the association between social support and undernutrition, and the mediating effect accounted for 29.67% of the total effect.

**Conclusion:**

Undernutrition among CUFY was relatively high in Siem Reap Province, Cambodia. Maternal depression and household food insecurity partially mediated the association between maternal social support and undernutrition. Providing postpartum workshops promotes awareness about depression, enhances social support, and reduces household food insecurity.

**Supplementary Information:**

The online version contains supplementary material available at 10.1186/s12889-025-25913-0.

## Introduction

Adequate nutrition is crucial to child development, particularly during the early years, from birth to 5 years of age. The absolute results of undernutrition are poor growth, infection and death, cognitive impairment, and low earning potential later in life [1]. Undernutrition is defined as insufficient intake of energy and nutrients to meet an individual’s needs to maintain good health [2]. Stunting is defined as a child’s height being significantly below the standard for their age [3]. Wasting is identified when a child’s weight is noticeably lower in proportion to their height [4, 5]. Undernutrition is the main global cause of poor health among children under five years old (CUFY) [6]. According to the joint data from UNICEF, the World Health Organization, and the World Bank, 27.4% of children under the age of five in Southeast Asia were stunted, whereas 8.2% were wasted [7]. Cambodia had a high prevalence of child undernutrition compared to the Southeast Asian countries [8]. Children’s malnutrition impacts the economy by 1.7% of GDP, equivalent to 266 million USD, with stunting alone costing 120 million USD in Cambodia [9]. According to the Cambodia Demographic Health Survey (CDHS), Cambodia was determined as the country with a significant public health concern for children’s health outcomes; the prevalence of stunting and wasting was 22% and 10% in 2021–2022, respectively [10]. From 2005 to 2022, the prevalence of wasting remained at approximately 10% [11, 12], highlighting a gap between the strategy and reality [13, 14] and the need to conduct research on the undernutrition among CUFY in Cambodia.

Maternal social support is a vital network of emotional, informational, and practical assistance that helps mothers navigate pregnancy and postpartum challenges, enhancing their mental health, strengthening parenting skills, and facilitating smoother transitions into parenthood [15, 16]. Maternal social support has long-term effects on children, including their birth weight and the risks of stunting or wasting [17]. Moreover, high maternal social support was more likely to feed a diverse diet to their children [18] and the prevention of wasting [19]. A study in Indonesia found that family support and social support were the determinants of stunting [20]. Families with strong family support have a 2.44 times higher likelihood of meeting good nutritional needs among CUFY [21]. Furthermore, a previous study among Hispanics in the US showed an association between household food insecurity and depression, with social support [22]. Conversely, no previous study examined whether household food insecurity and maternal depression mediate the effect of maternal social support in Cambodia.

Household food insecurity refers to a situation in which there is a lack of consistent access to food, which diminishes dietary quality, disrupts normal eating patterns, and can have negative consequences for nutrition, health, and well-being [23]. Household food insecurity was a significant determinant of children’s nutritional status in developing countries, where millions of households live in poverty and struggle to access nutritious food, posing a significant public health problem [24]. Children in households with food security face a significantly lower risk of stunting and wasting compared to children from households experiencing severe food insecurity [25, 26]. A study in Ethiopia found that 54% of families experienced moderate food insecurity. These families had a risk of stunting that was significantly two times higher. While wasting was not statistically significant, however increased risk was 1.4 times compared with food security [27]. A study in Rwanda found that moderate and severe household food insecurity were correlated with stunting and severe stunting [28]. Moreover, higher maternal social support was found to be a protective factor against household food insecurity in Iran [29]. Thus, household food insecurity may act as a mediator in the relationship between maternal social support and undernutrition among CUFY.

Maternal depression symptoms include depression, irritability, tiredness, sleeplessness, changes in appetite, inability to enjoy activities, loss of interest, negative thoughts, guilt, anxiety, avoidance of social interactions, hopelessness, thoughts of self-harm or suicide, and even psychosis in severe cases [30]. Maternal depression was associated with a significantly higher risk of stunting and wasting in children, with a crude odds ratio of 2.23 [31]. Children of depressed mothers were nearly three times more likely to be stunted compared to children of non-depressed mothers [32, 33]. A study conducted in Bangladesh similarly found that maternal depressive symptoms were significantly associated with a higher risk of stunting and wasting [34]. Maternal depression symptoms significantly contribute to child undernutrition by negatively affecting a mother’s sense of responsibility and parenting practices, ultimately hindering her ability to provide an adequate diet for her child [35]. A study in the United States of America revealed that household food insecurity predicted maternal depression [36]. Additionally, maternal depression was significantly associated with maternal social support and household food insecurity [37]. Another cross-sectional study found that maternal social support affected maternal depression and childbirth length [38]. Therefore, maternal depression may mediate the relationship between maternal social support and undernutrition among CUFY.

According to previous studies, household food insecurity and maternal depression may mediate the relationship between maternal social support and undernutrition among CUFY. Moreover, no studies explored the mediating effect of household food insecurity and maternal depression on the association between maternal social support and undernutrition among CUFY in Cambodia. Thus, this study aimed to clarify the chain mediating effect of household food insecurity and maternal depression in the relationship between maternal social support and undernutrition among CUFY in Cambodia.

## Method

### Study design and data collection

This study was a cross-sectional survey conducted in Siem Reap Province, Cambodia. Firstly, considering the influence of geographical location, one subordinate district in Siem Reap Province was selected randomly. In the second stage, three communes (Chreav, Chong Knies, and Siem Reap) were randomly chosen as the subsampling units from the first stage. In these communes, all mothers and CUFY constituted the total sample for this study. The questionnaire was designed in a semi-structured format for this study. Data collection was conducted with mothers of children under 5 years old, face-to-face, from August to September 2024. The sample size was calculated by the formula n = Z^2^p(1-p)/d^2^, where *Z* is the level of significance at the 95% CI (= 1.96), *d* is the desired degree of precision, typically set at 0.05, and *p* is the expected prevalence. The prevalence of stunting and wasting were 25.7% and 12.8% [10]. The total study sample size was set at a minimum of 450. Inclusion criteria included mothers/caregivers who were available for interviews in this study, mothers/caregivers who have children aged under 5, and children under 5 years old. Exclusion criteria were children who are more than 5 years old, mothers/caregivers who were unavailable for face-to-face interviews, and children who were ill.

### Measurement

#### Maternal social support

The Maternity Social Support Scale (MSSS) was used to measure mothers’ social support. The MSSS consisted of 6 questions, with responses measured using a 5-point Likert scale [39]. Women with scores in the highest tertile (≥ 24) were classified as having high social support. Those with scores ranging from 18 to 23 were classified as having medium social support, while scores below 18 indicated low social support. The MSSS was used Chinese translation by Li et al. (2020) [40], which had good reliability and validity, demonstrating a Cronbach’s α of 0.75.

#### Household food insecurity

The Household Food Insecurity Access Scale (HFIAS) is a continuous measure of the degree of food insecurity (access) in the household in the past four weeks (30 days). According to the categorization scheme recommended by the HFIAS Indicator Guide [41], the total scores were divided into four categories representing food security levels: (1) food security, (2) mild food insecurity, (3) moderate food insecurity, and (4) severe food insecurity. The HFIAS was used by Knueppel et al. (2010) [42], which demonstrated good validity and reliability, with a Cronbach’s α of 0.90 for measuring food insecurity.

#### Maternal depression

Maternal depression was measured by the Patient Health Questionnaire-2 (PHQ-2) scale. This scale is a brief screening tool used to identify potential cases of depression, focusing on the frequency of depressed mood and anhedonia (loss of interest or pleasure) over the past two weeks. The total score ranges from 0 to 6. A score of 3 is the optimal cut point to screen for depression symptoms. In this study, the Hong Kong version of the PHQ-2 questionnaire by Yu et al. (2011) was used [43], which demonstrated good reliability and validity with a Cronbach’s α of 0.76.

#### Child’s undernutrition

The indicator variables that constructed the latent variable of undernutrition among children under 5 years were stunting, set as height-for-age z-score (HAZ) below − 2 SD, and wasting, weight-for-height z-score (WHZ) below − 2SD [44].

### Statistical analysis

A descriptive analysis was conducted to show the prevalence of undernutrition (stunting and wasting) among CUFY. The chi-square test was initially performed to examine the associations between variables and outcomes of stunting and wasting. Pearson correlation analysis was applied to clarify the relationship between maternal social support, maternal depression, household food insecurity, stunting, and wasting. Structural equation modeling was used to analyze the chain mediating effect of household food insecurity and maternal depression on the relationship between maternal social support and a child’s undernutrition. The model fitness was assessed by the following indices: GFI >0.9, AGFI >0.9, CFI >0.9, NFI >0.9, IFI >0.9, RMSEA < 0.08 [45]. The structural equation model analysis was performed by AMOS version 27.0.

The Anthro WHO software was utilized to classify undernutrition outcomes, specifically identifying instances of stunting and wasting among CUFY. Data management was done using Microsoft Excel. Statistical analysis was conducted using the Statistical Package for the Social Sciences (SPSS), version 25.0. The results were considered significant at *P* < 0.05.

## Result

### Social demographic characteristics

Table 1 shows the demographic variables of the sample (*n* = 450). The study sample comprised children aged 12 to 23 months, making up 24% of the total sample, with boys representing 54.7% of the total sample. The majority, 69.8%, had normal birth weights, while 39.3% were firstborns. Severe household food insecurity was prevalent among 32.8% of the participants, 42.4% of mothers reported symptoms of depression, and 42.7% reported low levels of social support. Stunting was more prevalent among children under 12 months old, with a prevalence of 11.7%. Boys had a stunting prevalence of 21.6%, which was higher than that of girls. Stunting was also more prevalent in groups of low birthweights (20%), ≥5th child’s order (12.2%), severe household food insecurity (18.8%), maternal depression symptoms (24.5%), and low maternal social support (24.9%). Wasting was higher in children younger than 12 months old (6.9%) and boys (10.4%). Additionally, children with low birthweight (13.8%), those ≥ 5th birth order (6.1%), children living in severely food-insecure households (10.3%), those whose mothers suffered from maternal depression (10.9%), and those with mothers who had low maternal social support (12.9%) had a higher prevalence of wasting compared to other groups. The results revealed that stunting and wasting were statistically significant in age, sex, birth weight, children’s order, maternal social support, household food insecurity, and maternal depression.

**Table 1:** Description and univariate analysis of undernutrition (stunting and wasting) among children under 5 years in Siem Reap Province, Cambodia

Variable		Stunting			Wasting		
	Total	Yes	No	χ2/*P*	Yes	No	χ2/*P*
	*n*(%)	*n*(%)	*n*(%)		*n*(%)	*n*(%)	
Total	450(100)	157(34.9)	293(65.1)		70(15.6)	380(84.4)	
Age (months)							
< 12	104(23)	53(11.7)	51(11.3)	20.06^***^	31(6.9)	73(16.2)	32.21^***^
12–23	108(24.0)	34(7.6)	74(16.4)		21(4.7)	87(19.3)	
24–35	93(20.7)	35(7.8)	58(12.9)		12(2.7)	81(18.0)	
36–59	145(32.3)	35(7.8)	110(24.5)		6(1.3)	139(30.9)	
Sex							
Boy	246(54.7)	97(21.6)	149(33.1)	4.93^*^	47(10.4)	199(44.2)	
Girl	204(45.3)	60(13.3)	144(32)		23(5.2)	181(40.2)	5.21^*^
Birthweight							
Low	119(26.4)	90(20.0)	29(6.5)	118.21^***^	62(13.8)	57(12.6)	
Normal and High	331(73.6)	67(14.9)	264(58.7)		8(1.8)	323(71.8)	164.49^***^
Child Order							
1st	177(39.3)	33(7.3)	144(32.0)	56.07^***^	18(4.0)	159(35.3)	
2nd	113(25.1)	36(8.1)	77(17.1)		10(2.2)	103(22.9)	25.45^***^
3rd-4th	74(16.5)	33(7.3)	41(9.1)		15(3.3)	59(13.1)	
≥ 5th	86(19.1)	55(12.2)	31(6.9)		27(6.1)	59(13.1)	
HFI							
Food secure	156(34.7)	16(3.6)	140(31.1)	80.24^***^	4(0.9)	152(33.7)	
Mild insecure	71(15.8)	21(4.7)	50(11.1)		10(2.2)	61(13.6)	47.60^***^
Moderate insecure	75(16.7)	35(7.8)	40(8.9)		10(2.2)	65(14.4)	
Severe insecure	148(32.8)	85(18.8)	63(14.0)		46(10.3)	102(22.7)	
MD							
No	259(57.5)	47(10.4)	212(47.1)	75.29^***^	21(4.7)	238(52.8)	
Yes	191(42.4)	110(24.5)	81(18.0)		49(10.9)	142(31.6)	25.76^***^
MSS							
Low	192(42.7)	112(24.9)	80(17.8)	87.65^***^	58(12.9)	134(29.7)	
Medium	127(28.2)	32(7.1)	95(21.1)		7(1.6)	120(26.7)	54.88^***^
High	131(29.1)	13(2.9)	118(26.2)		5(1.1)	126(28.0)	

*HFI* Household food insecurity, *MD* Maternal depression, *MSS* Maternal social support

*P < 0.05, **P < 0.01, ***P < 0.001; low birthweight < 2500 g, normal birthweight: 2500–4000 g, high birthweight > 4000 g

### Correlation between variables

Table 2 shows the correlation matrix for the key variables of the study. All independent variables were statistically significantly associated with stunting and wasting. Maternal social support (*r*=-0.417, *P* < 0.01 for stunting, *r*=-0.246, *P* < 0.01 for wasting) was positively correlated. Household food insecurity (*r* = 0.399, *P* < 0.01 for stunting, *r* = 0.291, *P* < 0.01 for wasting) and maternal depression (*r* = 0.443, *P* < 0.01 for stunting, *r* = 0.267, *P* < 0.01 for wasting) were negatively correlated.

**Table 2:** Bivariate correlation analysis between maternal social support, household food insecurity, maternal depression, stunting, and wasting

	MSS	HFI	MD	Stunting	Wasting
MSS	1				
HFI	− .332^a**^	1			
MD	− .473^a**^	.337^a**^	1		
Stunting	− .417^b**^	.399^b**^	.443^b**^	1	
Wasting	− .246^b**^	.291^b**^	.267^b**^	.342^b**^	1

*HFI* Household food insecurity, *MD* Maternal depression, *MSS* Maternal social support

^a^Pearson correlation analysis, ^b^Chi-square test

### Mediation effect analysis

Table 3 shows the fitness indices of the hypothetical model in this study. The fitness indices demonstrated that the model had a good fit (GFI = 0.933, AGFI = 0.901, CFI = 0.905, NFI = 0.962, IFI = 0.977, RMSEA = 0.056), implying that empirical data fit the hypothesized model well.

**Table 3:** Model fitness indices

	GFI	AGFI	CFI	NFI	IFI	RMSEA
Suggest value	> 0.90	> 0.90	> 0.90	> 0.90	> 0.90	< 0.08
Actual value	0.933	0.901	0.977	0.962	0.978	0.056
Decision	Good fit	Good fit	Good fit	Good fit	Good fit	Good fit

*CMIN* Chi-square, *DF* Degree of freedom, *GFI* The goodness-of-fit index, *AGFI* The adjusted goodness-of-fit index, *IFI* The incremental fit index, *CFI* The comparative fit index, *RMSEA * Root mean square error of approximation

**Fig. 1:**
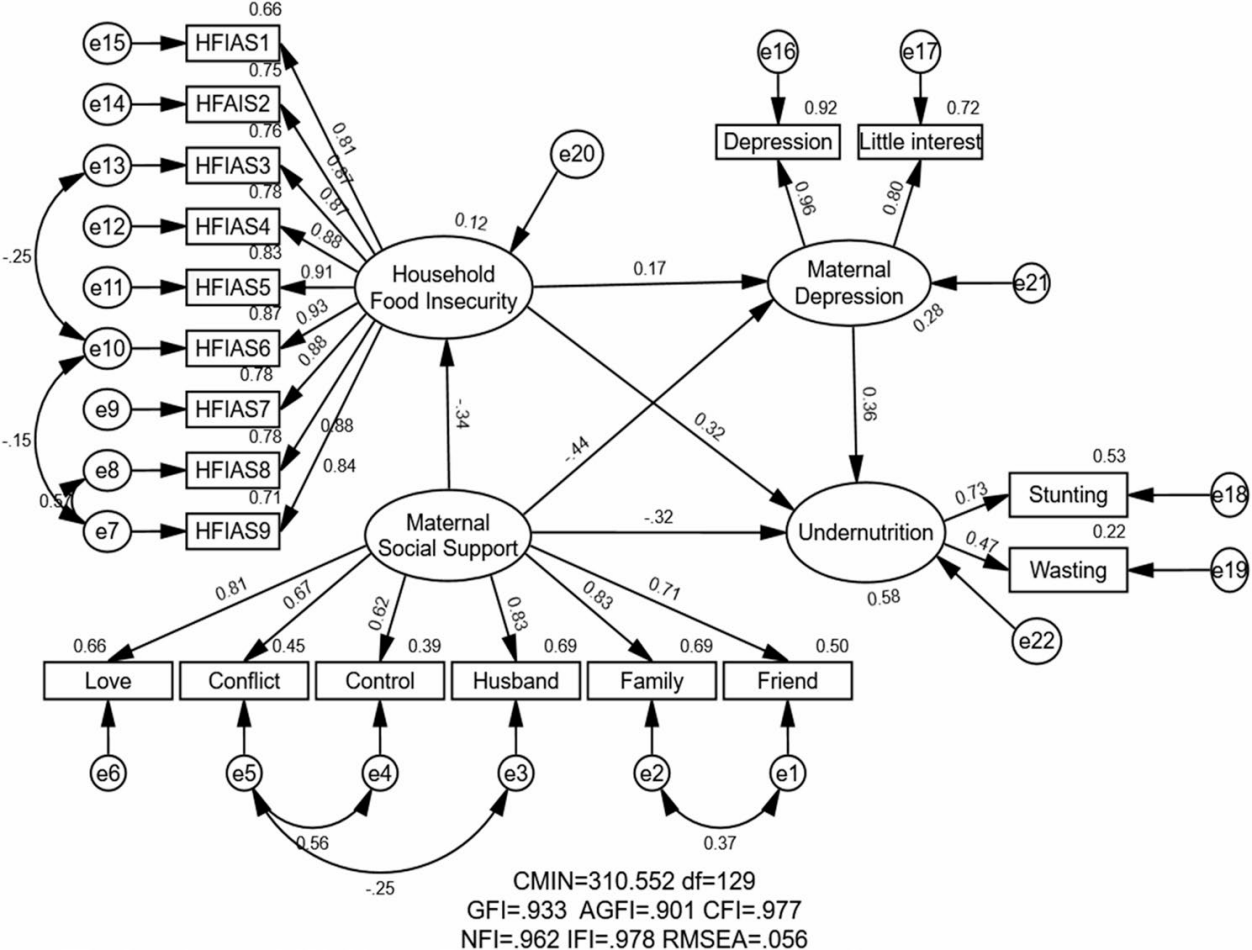
Association between maternal social support and undernutrition among CUFY: the mediating role of maternal depression and household food insecurity

Figure 1 illustrated the results of the result of the structural equational modeling analysis of the relationship between maternal social support and child’s undernutrition via the household food insecurity and maternal depression. Maternal social support exerted a direct negative effect and an indirect negative effect on undernutrition through two mediating pathways (household food insecurity and maternal depression). The one direct effect from maternal social support on undernutrition was clearly demonstrated in Fig. 1, while three indirect effects from maternal social support on undernutrition were as follow: (1) maternal social support ➔ household food insecurity➔undernutrition; (2) maternal social support ➔maternal depression ➔ undernutrition; (3) maternal social support➔household food insecurity➔ maternal depression➔ undernutrition. In this study, the mediating effect was demonstrated by a 95% CI of the indirect path coefficient excluding zero.

The standardized direct effect of maternal social support on children’s undernutrition among CUFY was $\:\beta\:$= -0.32 (95% Cl = 0.178 to 0.450), indicating that women who had a higher score of social support had a lower risk of their children’s undernutrition compared with those who had lower social support. The standardized effect of maternal social support on children’s undernutrition via household food insecurity was $\:\beta\:$ = -0.051 (with a mediating effect of 11.2%), implying that women with higher social support had a lower risk of their children’s undernutrition via household food insecurity. The standardized effect of maternal social support on children’s undernutrition through maternal depression was $\:\beta\:=$-0.074 (with a mediating effect of 16.26%), indicating that women with higher social support had a lower risk of the children’s undernutrition due to reduced maternal depression. Furthermore, the standardized effect of social support on children’s undernutrition through the combined mediation of household food insecurity and maternal depression was $\:\beta\:=$-0.010 (accounting for 2.19% of the total effect), implying that women with higher social support had a lower risk of their children’s undernutrition via reduced household food insecurity and maternal depression. As shown in Table 4, the total mediating effect of maternal social support on undernutrition among CUFY was $\:\beta\:=$-0.135 (95% Cl = 0.020 to 0.099), accounting for 29.67%.

**Table 4:** Mediation effects of undernutrition in structural equation modeling

Model Pathway	$\:\beta\:$	SE	95%CI		Percent
			LLCL	ULCL	(%)
Total effect	-0.455	0.034	0.218	0.353	100
Direct effect	-0.320	0.070	0.178	0.450	70.33
MSS–>Undernutrition					
Indirect effect	-0.135	0.020	0.099	0.182	29.67
MSS–>HFI–>Undernutrition	-0.051				11.20
MSS–>MD–>Undernutrition	-0.074				16.26
MSS–>HFI–>MD–>Undernutrition	-0.010				2.19

*SE* Standard error, *CI * Confidence interval; *LLCL * Lower limits confidence interval, *ULCL * Upper limits confidence interval, *HFI * Household food insecurity, *MD * Maternal depression, *MSS * Maternal social support

## Discussion

### Finding compared with previous studies

#### Prevalence of undernutrition in CUFY

In the present study, the prevalence of stunting and wasting among CUFY was 34.9% and 15.6%, respectively. A previous study in Cambodia found the prevalence of stunting at 32% and wasting at 8% among children aged 2–5 years in households with and without home gardens [46]. The difference may be because wasting was more prevalent among children from 0 to 2 years old [47]. Moreover, stunting and wasting were found to be statistically significant with respect to age, birth weight, and children’s order in this study, consistent with findings from previous studies conducted separately in Ethiopia [48], Zambia [49], and Ghana [50].

#### Relationship between social support and undernutrition of CUFY

SEM results found that maternal social support was negatively correlated with undernutrition among CUFY, implying that higher maternal social support is associated with a lower risk of undernutrition. This suggested that a strong maternal social support network would benefit children’s nutrition, potentially leading to reduced stunting and improved overall child health outcomes [51]. Our result were consistent with a previous study in southeastern Brazil, which also found that higher social support was associated with a reduced risk of malnutrition [52].

#### Association between household food insecurity and undernutrition among CUFY

Household food insecurity was found to be an immediate factor influencing undernutrition among CUFY. In detail, this study found a positive relationship between household food insecurity and undernutrition. This result was similar to a previous study in Malaysia, which revealed that household food insecurity was a risk factor for wasting and stunting [53]. Moreover, a study in Tanzania revealed that household food security, measured by household energy adequacy per adult equivalent and household dietary diversity score, was inversely associated with undernutrition among adolescents. This implies that household food insecurity was positively correlated with undernutrition, a finding consistent with that of our study [54].

#### Relationship between maternal depression and undernutrition among CUFY

Our result was similar to that of a study in Ethiopia, which found that maternal depression symptoms were a risk factor for stunting in infants aged 5–10 months in northern Ethiopia [55]. Moreover, a previous study also found that current maternal depression was a significant risk factor for malnutrition among children in Selangor, Malaysia [56]; mothers with depressive symptoms were more than three times as likely to have stunted and underweight children compared to mothers without depression [57].

#### Relationship between the maternal social support and household food insecurity of CUFY

Moreover, the study found that maternal social support was negatively associated with household food insecurity. This finding indicates that maternal social support plays a crucial role in buffering households against food insecurity by providing social resources that help mothers manage food resources more effectively [58]. Our result was consistent with a previous study conducted in rural households of Nicaragua, which showed that perceived social support was a protective factor against food insecurity after adjusting for confounding factors [59], as well as another study in the Vakinankaratra Region, Madagascar, which found that mothers with optimal social support had lower household food insecurity in the adjusted models [60].

#### Association between household food insecurity and maternal depression of CUFY

Household food insecurity and maternal depression were positively correlated in our study, indicating that higher household food insecurity would predict higher depression among mothers. Our result was consistent with a previous study among pregnant women in Ethiopia, which found that higher baseline food insecurity were positively and significantly associated with perinatal depression [61]. Another study in rural families in the USA further found a bidirectional relationship between household food insecurity and maternal depression [62].

#### Association between the maternal social support and undernutrition via household food insecurity and maternal depression of the CUFY

This study demonstrated that household food insecurity and maternal depression mediated the relationship between maternal social support and undernutrition among CUFY in Cambodia. However, a previous study found that household food insecurity mediated the relationship between maternal social support and child nutritional status in Peru, Ethiopia, Vietnam, and India [63]. Moreover, another study found that maternal depression mediated the relationship between maternal social support and undernutrition in Indonesia [64].

The chain mediation effect of household food insecurity and maternal depression on the relationship between maternal social support and undernutrition could be interpreted as follows. During the transition to motherhood, women often experience a decline in social interactions and find it increasingly difficult to maintain connections with friends, which may lead to a decrease in their social activities [65]. Moreover, the transition to parenthood was associated with changes in marital satisfaction, negative communication patterns, and poor conflict management [66], which may further exert a negative effect on maternal social support. Lower maternal social support could in turn, lead to a higher prevalence of household food insecurity [67] and maternal depression [68]. Furthermore, household food insecurity [69] and maternal depression [70] might finally contribute to undernutrition among CUFY.

### Implications

Firstly, the government’s strategy should include maternal peer support programs to educate family, friends, and husbands about postpartum support, thereby enhancing maternal social support and helping lower undernutrition of CUFY. Secondly, early screening of antenatal and postpartum depression, along with increasing awareness of depression symptoms, should be implemented to address maternal mental health and its impact on child nutrition. Thirdly, household food security should be promoted through community nutrition education, raising farming awareness and support for organic and safe chemical farming via multisectoral coordination, all of which would contribute to reducing undernutrition among CUFY.

### Limitations

This study had several limitations. First, there may be other underlying factors that influence the association between maternal social support and undernutrition beyond household food insecurity and maternal depression, which require further research to clarify. Second, this study only focused on Siem Reap Province in Cambodia; further research is needed to be conducted in other regions with a larger sample size. Third, data collection was conducted during Cambodia’s rainy season (August–September), so the observed prevalence of undernutrition of CUFY may have been affected by seasonal factors such as fluctuations in food availability, patterns of illness, and increased family workloads.

## Conclusion

This study found that the prevalence of undernutrition among children under 5 years in Siem Reap province remains high. The results of the SEM showed that maternal social support directly influenced undernutrition and also had an indirect effect through household food insecurity and maternal depression. Future research on interventions should focus on reducing undernutrition through improving maternal social support, addressing household food insecurity, and managing maternal depression among households with CUFY.

## Supplementary Information

Supplementary Material 1.

## Data Availability

Data can be obtained from the corresponding author and will be provided upon request.

## References

[1] UNICEF. The state of the world’s children 2019. Children. Food and Nutrition: Growing well in a changing world; 2019.

[2] Maleta K. *Undernutrition.* Malawi medical journal. J Med Association Malawi. 2006;18(4):189. PMC3345626 27529011

[3] De Onis M, Branca F. Childhood stunting: a global perspective. Matern Child Nutr. 2016;12:12–26. 27187907 10.1111/mcn.12231 PMC5084763

[4] Kerac M, et al. Prevalence of wasting among under 6-month-old infants in developing countries and implications of new case definitions using WHO growth standards: a secondary data analysis. Arch Dis Child. 2011;96(11):1008–13. 21288999 10.1136/adc.2010.191882 PMC3195296

[5] De Onis M, et al. Prevalence thresholds for wasting, overweight and stunting in children under 5 years. Public Health Nutr. 2019;22(1):175–9. 30296964 10.1017/S1368980018002434 PMC6390397

[6] Organization WH. Levels and trends in child malnutrition child malnutrition: UNICEF/WHO/World bank group joint child malnutrition estimates: key findings of the 2023 edition. World Health Organization; 2023.

[7] UNICEF. Southeast Asia regional report on maternal nutrition and complementary feeding. UNICEF East Asia and the Pacific Regional Office; 2021.

[8] FAO, UNICEF, WFP, WHO. Asia and the Pacific Regional Overview of Food Security and Nutrition 2020: Maternal and child diets at the heart of improving nutrition. Bangkok, FAO. 2021. 10.4060/cb2895en.

[9] Moench-Pfanner R, et al. The economic burden of malnutrition in pregnant women and children under 5 years of age in Cambodia. Nutrients. 2016;8(5):292. 27187462 10.3390/nu8050292 PMC4882705

[10] National Institute of Statistics, Ministry of Health, and, Program TDHS. Cambodia demographic and health survey 2021–22. and ICF: Phnom Penh, Cambodia, and Rockville, Maryland, USA: NIS; 2022. MoH.

[11] Health NIoP, Sthiti VJ, Macro O. Cambodia demographic and health survey, 2005. National Institute of Public Health and National Institute of Statistics; 2006.

[12] Rao N, Pearson E. An evaluation of early childhood care and education programmes in Cambodia. Unpublished manuscript. 2007. http://www.unicef.org/evaldatabase/files/CBD_early_childhoodcare_evaluation.pdf.

[13] Van Beekum M, et al. The associations between stunting and wasting at 12 months of age and developmental milestones delays in a cohort of Cambodian children. Sci Rep. 2022;12(1):17859. 36284133 10.1038/s41598-022-22861-2 PMC9596435

[14] Mutunga M, et al. The relationship between wasting and stunting in Cambodian children: secondary analysis of longitudinal data of children below 24 months of age followed up until the age of 59 months. PLoS ONE. 2021;16(11):e0259765. 34794170 10.1371/journal.pone.0259765 PMC8601787

[15] United Nations Children’s Fund (UNICEF). Improving Maternal Nutrition: An Acceleration Plan to Prevent Malnutrition and Anaemia during Pregnancy (2024–2025). UNICEF, New York. 2024.

[16] Saleh A, et al. Role of maternal in preventing stunting: a systematic review. Gac Sanit. 2021;35:S576–82. 34929905 10.1016/j.gaceta.2021.10.087

[17] Imanishi Y, et al. The association between maternal social support levels during pregnancy and child development at three years of age: the Japan environment and children’s study. Environ Health Prev Med. 2024;29:18–18. 38508729 10.1265/ehpm.23-00211 PMC10965412

[18] Matare C, et al. Maternal Decision-Making Autonomy, mental Health, gender norm Attitudes, and social support during pregnancy predict child Care-Giving and stunting in rural Zimbabwe. Curr Developments Nutr. 2020;4:nzaa053071.

[19] Nugraha SY, et al. Influence of social support for families parenting mother in preventing child stunting. Int J Nurs Midwifery Sci (IJNMS). 2019;3(3):122–6.

[20] Nugroho E, et al. Social determinants of stunting in Indonesia. Jurnal Kesehatan Masyarakat. 2023;18(4):546–55.

[21] Wiliyanarti PF, Wulandari Y, Nasrullah D. Behavior in fulfilling nutritional needs for Indonesian children with stunting: related culture, family support, and mother’s knowledge. J Public Health Res. 2022;11(4):22799036221139938. 36531594 10.1177/22799036221139938 PMC9755554

[22] Kapulsky L, Tang AM, Forrester JE. Food insecurity, depression, and social support in HIV-infected Hispanic individuals. J Immigr Minor Health. 2015;17:408–13. 25047405 10.1007/s10903-014-0076-x PMC4303548

[23] Smith M, et al. Household food security: concepts and definitions: an annotated bibliography. Volume 8. Sussex: Institute of Development Studies Brighton; 1993.

[24] Saaka M, Osman SM. Does household food insecurity affect the nutritional status of preschool children aged 6–36 months? Int J Popul Res. 2013;2013(1):304169.

[25] Motbainor A, Worku A, Kumie A. Stunting is associated with food diversity while wasting with food insecurity among underfive children in East and West Gojjam zones of Amhara Region, Ethiopia. PLoS ONE. 2015;10(8):e0133542. 26285047 10.1371/journal.pone.0133542 PMC4540277

[26] Betebo B, et al. Household food insecurity and its association with nutritional status of children 6–59 months of age in East Badawacho District, South Ethiopia. J Environ Public Health. 2017;2017(1):6373595. 28408936 10.1155/2017/6373595 PMC5376409

[27] Abdurahman AA, et al. Household food insecurity May predict underweightand wasting among children aged 24–59 months. Ecol Food Nutr. 2016;55(5):456–72. 27467901 10.1080/03670244.2016.1207069

[28] Agho KE, et al. Moderate and severe household food insecurity predicts stunting and severe stunting among Rwanda children aged 6–59 months residing in gicumbi district. Maternal & child nutrition; 2019;15(3): e12767. 10.1111/mcn.12767 PMC7198954 30548790

[29] Sharifi N et al. The relationship between social support and food insecurity in pregnant women: A Cross-sectional study. J Clin Diagn Res. 2017;11(11). 10.7860/JCDR/2017/29987.10858.

[30] Liu X, Wang S, Wang G. Prevalence and risk factors of postpartum depression in women: a systematic review and meta-analysis. J Clin Nurs. 2022;31(19–20):2665–77. 34750904 10.1111/jocn.16121

[31] Ashaba S, et al. Maternal depression and malnutrition in children in Southwest uganda: a case control study. BMC Public Health. 2015;15:1–6. 26712120 10.1186/s12889-015-2644-y PMC4693407

[32] Wemakor A, Mensah KA. Association between maternal depression and child stunting in Northern ghana: a cross-sectional study. BMC Public Health. 2016;16:1–7. 27557725 10.1186/s12889-016-3558-z PMC4997709

[33] Apriliana T, et al. A contributing factor of maternal pregnancy depression in the occurrence of stunting on toddlers. J Public Health Res. 2022;11(2):pjphr20212738. 10.4081/jphr.2021.2738 PMC8941310 35244359

[34] Karim KMR, et al. Child undernutrition is associated with maternal mental health and other sociodemographic factors in low-income settings in Dhaka, Bangladesh. PLoS ONE. 2025;20(5):e0322507. 40315185 10.1371/journal.pone.0322507 PMC12047756

[35] Santos IS, et al. Long-lasting maternal depression and child growth at 4 years of age: a cohort study. J Pediatr. 2010;157(3):401–6. 20400093 10.1016/j.jpeds.2010.03.008 PMC2937222

[36] Nagata JM, et al. Food insecurity is associated with maternal depression and child pervasive developmental symptoms in low-income Latino households. J Hunger Environ Nutr. 2019;14(4):526–39. 31673300 10.1080/19320248.2018.1434101 PMC6822564

[37] Wemakor A, Bukari M, Atariba R. Household food insecurity, low maternal social support and maternal common mental disorders in East mamprusi Municipality, Ghana. BMC Public Health. 2023;23(1):1255. 37380991 10.1186/s12889-023-16157-x PMC10308758

[38] Elsenbruch S, et al. Social support during pregnancy: effects on maternal depressive symptoms, smoking and pregnancy outcome. Hum Reprod. 2007;22(3):869–77. 17110400 10.1093/humrep/del432

[39] Webster J, et al. Measuring social support in pregnancy: can it be simple and meaningful? Birth. 2000;27(2):97–101. 11251486 10.1046/j.1523-536x.2000.00097.x

[40] Li G, et al. Reliability and validity of the Chinese version of maternity social support scale. Sichuan Mental Health. 2020;33(3):268.

[41] Coates J, Swindale A, Bilinsky P. Household Food Insecurity Access Scale (HFIAS) for measurement of food access: indicator guide: version 3. 2007.

[42] Knueppel D, Demment M, Kaiser L. Validation of the household food insecurity access scale in rural Tanzania. Public Health Nutr. 2010;13(3):360–7. 19706211 10.1017/S1368980009991121

[43] Yu X, et al. Screening for depression with the patient health Questionnaire-2 (PHQ-2) among the general population in Hong Kong. J Affect Disord. 2011;134(1–3):444–7. 21665288 10.1016/j.jad.2011.05.007

[44] Duggan M. Anthropometry as a tool for measuring malnutrition: impact of the new WHO growth standards and reference. Ann Trop Paediatr. 2010;30(1):1–17. 20196929 10.1179/146532810X12637745451834

[45] Sathyanarayana S, Mohanasundaram T. Fit indices in structural equation modeling and confirmatory factor analysis: reporting guidelines. Asian J Econ Bus Acc. 2024;24(7):561–77.

[46] Sek L et al. Dietary diversity and nutritional status of 2 to 5 years old children in households with and without home gardens in selected districts in Siem reap province, Cambodia. Malaysian J Nutr, 27(2): 209-219, 2021

[47] Karlsson O et al. Child wasting before and after age two years: a cross-sectional study of 94 countries. EClinical Medicine. 2022;46. 10.1016/j.eclinm.2022.101353. 10.1016/j.eclinm.2022.101353 PMC8961190 35360149

[48] Kassie GW, Workie DL. Determinants of under-nutrition among children under five years of age in Ethiopia. BMC Public Health. 2020;20:1–11. 32220224 10.1186/s12889-020-08539-2 PMC7099779

[49] Musenge EM, et al. Prevalence and determinants of malnutrition among under-five children in Lusaka urban, Zambia. Tanzan J Health Res. 2019;21(1):1–13.

[50] Boah M, et al. The epidemiology of undernutrition and its determinants in children under five years in Ghana. PLoS ONE. 2019;14(7):e0219665. 31365528 10.1371/journal.pone.0219665 PMC6668784

[51] Mumpuningtias ED, Hidayat S, Kurniawan AW. Maternal and child factors of stunted children: a case control study. Int J Public Health. 2025;14(2):852–9.

[52] de Carvalhaes BL, Benício MAMHDA, Barros AJ. Social support and infant malnutrition: a case–control study in an urban area of southeastern Brazil. Br J Nutr. 2005;94(3):383–9. 16176609 10.1079/bjn20051505

[53] Naser IA, et al. Association between household food insecurity and nutritional outcomes among children in Northeastern of Peninsular Malaysia. Nutr Res Pract. 2014;8(3):304–11. 24944776 10.4162/nrp.2014.8.3.304 PMC4058565

[54] Cordeiro LS, et al. Household food security is inversely associated with undernutrition among adolescents from Kilosa, Tanzania. J Nutr. 2012;142(9):1741–7. 22810984 10.3945/jn.111.155994

[55] Anato A, et al. Maternal depression is associated with child undernutrition: A cross-sectional study in Ethiopia. Matern Child Nutr. 2020;16(3):e12934. 31833231 10.1111/mcn.12934 PMC7296785

[56] Din MAC, Teng NIMF, Manaf ZA. Is maternal depression a risk factor for malnutrition among Malay children? A Case-Control study in Selangor, Malaysia. Malaysian J Med Health Sci. 2021;17(3).10.1177/1367493517721063.

[57] Saeed Q, et al. Maternal depressive symptoms and child nutritional status: A cross-sectional study in socially disadvantaged Pakistani community. J Child Health Care. 2017;21(3):331–42. 29119823 10.1177/1367493517721063

[58] Mokari-Yamchi A, et al. Food security and its association with social support in the rural households: a cross-sectional study. Prev Nutr Food Sci. 2020;25(2):146. 32676465 10.3746/pnf.2020.25.2.146 PMC7333000

[59] Schmeer KK, et al. Maternal resources and household food security: evidence from Nicaragua. Public Health Nutr. 2015;18(16):2915–24. 25563386 10.1017/S1368980014003000 PMC10271382

[60] Komakech J, et al. Maternal social support is associated with child complementary Feeding, and household food security in the Vakinankaratra Region, Madagascar. Curr Developments Nutr. 2020;4:nzaa053060.

[61] Biratu A, et al. Food insecurity and perinatal depression among pregnant women in BUNMAP cohort in ethiopia: a structural equation modelling. Public Health Nutr. 2024;27(1):e120. 38605538 10.1017/S1368980024000855 PMC11075105

[62] Huddleston-Casas C, Charnigo R, Simmons LA. Food insecurity and maternal depression in rural, low-income families: a longitudinal investigation. Public Health Nutr. 2009;12(8):1133–40. 18789167 10.1017/S1368980008003650

[63] Lee H-Y, Song IH, Kawachi I. Maternal and child social support and food availability in relation to child growth in four low-and middle-income countries. Sci Rep. 2022;12(1):5910. 35396562 10.1038/s41598-022-09850-1 PMC8993861

[64] Nurfurqoni FA et al. Family function, social support, postpartum depression, and maternal parenting practices: Their impact on infant growth. in BIO Web of Conferences. EDP Sciences. 2025.

[65] Rözer JJ, Poortman A-R, Mollenhorst G. The timing of parenthood and its effect on social contact and support. Demographic Res. 2017;36:1889–916.

[66] Rhoades Galena K, Scott MS, Markman J, Howard. The effect of the transition to parenthood on relationship quality: an eight-year prospective study. J Personal Soc Psychol. 2009;96(3):601–19. 10.1037/a0013969 PMC2702669 19254107

[67] Nagata JM, et al. Around the table: food insecurity, socioeconomic status, and instrumental social support among women living in a rural Kenyan Island community. Ecol Food Nutr. 2015;54(4):358–69. 25680030 10.1080/03670244.2014.995790 PMC4466072

[68] Hajipoor S, et al. The relationship between social support and postpartum depression. J Holist Nurs Midwifery. 2021;31(2):93–103.

[69] Belayneh M, Loha E, Lindtjørn B. Seasonal variation of household food insecurity and household dietary diversity on wasting and stunting among young children in a drought prone area in South ethiopia: a cohort study. Ecol Food Nutr. 2021;60(1):44–69. 32672490 10.1080/03670244.2020.1789865

[70] Khan AM. Maternal mental health and child nutritional status in an urban slum in bangladesh: A cross-sectional study. PLOS Global Public Health. 2022;2(10):e0000871. 36962625 10.1371/journal.pgph.0000871 PMC10021263

