# Supplementary material for: Relationship between maternal social support and undernutrition among children under 5 years in Siem Reap Province, Cambodia: the chain mediating roles of household food insecurity and maternal depression
Source: BMC Public Health. 2025 Dec 11;26:802. doi: 10.1186/s12889-025-25913-0 (PMC12964837; doi:10.1186/s12889-025-25913-0)
Supplement: Supplementary file 1 — Supplementary Material 1. [file 12889_2025_25913_MOESM1_ESM.pdf]

Questionnaire

"Association between maternal social support and child's undernutrition among children under 5 years in Siem Reap Province, Cambodia: the chain mediating effect of household food insecurity and maternal depression"

Submission ID: 24693868-c26f-484f-af0a-b6070ad87633

1. Children's characteristics

Children's age……………years

Gender: □ Boy □Girl

Birth weight: …………...Kg

Birth order: □ 1st, □2nd □ 3rd □ 4th □ 5th □ 6th

Children height: ……………...cm

Children weight ………………Kg

2. Household Food Insecurity

2.1 In the past four weeks, did you worry that your household would not have enough food?

□ No (skip to Q2)
□ Rarely (1 or 2 in the past four weeks)
□ Sometimes (3-10 times in the past four weeks)
□ Often (more than ten times in the past four weeks)

2.2 In the past four weeks, were you or any household member not able to eat the kinds of foods you preferred because of a lack of resources?

□ No (skip to Q3)
□ Rarely (1 or 2 in the past four weeks)
□ Sometimes (3-10 times in the past four weeks)
□ Often (more than ten times in the past four weeks)

2.3 In the past four weeks, did you or any household member have to eat a limited variety of foods due to a lack of resources?

□ No (skip to Q4)
□ Rarely (1 or 2 in the past four weeks)
□ Sometimes (3-10 times in the past four weeks)

- Often (more than ten times in the past four weeks)

2.4 In the past four weeks, did you or any household member have to eat some foods you did not want because of a lack of resources to obtain other types of food?

- No (skip to Q5)
- Rarely (1 or 2 in the past four weeks)
- Sometimes (3-10 times in the past four weeks)
- Often (more than ten times in the past four weeks)

2.5 In the past four weeks, did you or any household member have to eat a smaller meal than you felt you needed because there was insufficient food?

- No (skip to Q6)
- Rarely (1 or 2 in the past four weeks)
- Sometimes (3-10 times in the past four weeks)
- Often (more than ten times in the past four weeks)

2.6 In the past four weeks, did you or any other household member have to eat fewer meals in a day because there was not enough food?

- No (skip to Q7)
- Rarely (1 or 2 in the past four weeks)
- Sometimes (3-10 times in the past four weeks)
- Often (more than ten times in the past four weeks)

2.7 In the past four weeks, was there ever no food to eat of any kind in your household because of a lack of resources to get food?

- No (skip to Q8)
- Rarely (1 or 2 in the past four weeks)
- Sometimes (3-10 times in the past four weeks)
- Often (more than ten times in the past four weeks)

2.8 In the past four weeks, did you or any household member sleep hungry at night because there was not enough food?

- No (skip to Q9)
- Rarely (1 or 2 in the past four weeks)
- Sometimes (3-10 times in the past four weeks)
- Often (more than ten times in the past four weeks)

2.9 In the past four weeks, did you or any household member go a whole day and night without eating anything because there was not enough food?

- No

□ Rarely (1 or 2 in the past four weeks)
□ Sometimes (3-10 times in the past four weeks)
□ Often (more than ten times in the past four weeks)

3. Patient Questionnaire-2 (PHQ-2)

Over the last 2 weeks, how often have you been bothered by the following all of these problems?

3.1 Do you feel little interest or pleasure in doing things?
□ Not at all □ several days □ more than half a day □ nearly every day

3.2 Do you feel down, depressed, or hopeless?
□ Not at all □ several days □ more than a half day □ nearly every day

4. Maternity Social Support Scale (MSSS)

Items	Always	Usually	Sometime	Rarely	Never
A. I have good friends who support me					
B. My family is always there for me.					
C. My husband/partner helps me a lot.					
D. There is a conflict with my husband/partner					
E. I feel controlled by my husband/partner.					
F. I feel loved by my husband/partner.					
